# The role of caspase-dependent and caspase-independent pathways of apoptosis in the premature rupture of the membranes: A case-control study

**DOI:** 10.18502/ijrm.v13i6.7285

**Published:** 2020-06-30

**Authors:** Ketut Surya Negara, Ketut Suwiyoga, Raka Sudewi, Nyoman Mantik Astawa, Gusti Nyoman Kamasan Arijana, Ketut Tunas, Tjokorda Gede Astawa Pemayun

**Affiliations:** ^1^Department of Obstetrics and Gynecology, Medical Faculty of Udayana University, Sanglah Hospital, Bali, Indonesia.; ^2^Department of Neurology, Medical Faculty of Udayana University, Sanglah Hospital Bali, Indonesia.; ^3^Laboratory of Veterinary Medicine, Udayana University Bali, Indonesia.; ^4^Biomedic Laboratory of Medical Faculty Udayana University, Bali, Indonesia.; ^5^Department of Public Health, Dhyana Pura University Bali, Indonesia.

**Keywords:** Premature, Membrane, Apoptosis, Caspase, Pregnancy.

## Abstract

**Background:**

Premature rupture of membrane (PROM) remains a problem in obstetrics, the mechanisms of PROM have not been clearly defined. Apoptosis is thought to play a key role in the mechanism, via caspase-dependent and caspase-independent pathways. Caspase-3, Apoptosis-inducing factor (AIF), and anti-apoptosis B-cell lymphoma 2 (Bcl-2) are hypothesized to be involved in PROM.

**Objective:**

To determine the role of caspase-dependent and caspase-independent pathways in the mechanism of PROM.

**Materials and Methods:**

This was a case-control study involving 42 pregnant women with gestational age between 20-42 wk. Participants were divided into the case group (with PROM) and control group (without PROM). Amniotic membranes were collected immediately after the delivery, and samples were taken from the site of membrane rupture. Immunohistochemical examination was done to determine the expression of Caspase-3, AIF, and Bcl-2.

**Results:**

The expressions of Caspase-3 (OR = 9.75; 95% CI = 2.16-43.95; p = 0.001) and AIF (OR = 6.60; 95% CI = 1.48-29.36; p = 0.009) were significantly increased, whereas, Bcl-2 expressions (OR = 8.00; 95% CI = 1.79-35.74; p = 0.004) were significantly decreased in the case group.

**Conclusion:**

High Caspase-3, AIF, and low Bcl-2 expression were the risk factors for PROM. Thus, it is evident that caspase-dependent and caspase-independent pathways are involved in the mechanism of PROM.

## 1. Introduction

Maternal and neonatal-related health problems are serious issues in Indonesia as demonstrated by high maternal and neonatal mortality rate, mostly caused by infection, hemorrhage, preeclampsia, and prematurity. Premature rupture of membrane (PROM) predisposes patients to infection, and its occurrence correlates with the increasing rates of maternal and perinatal morbidity and mortality.

PROM affects approximately 10-12% of all pregnancies, varying from 6 to 19% of term pregnancies and 6 to 8% of preterm pregnancies. Almost 80% of the cases occur in term pregnancies (1, 2). In a study conducted at a tertiary teaching hospital in Indonesia, PROM was found in 14.62% of all deliveries; 84.43% occurred in term and 15.57% in preterm pregnancies (3). The etiology of PROM is multi-factorial and the mechanisms are still unclear. The weakening of the extracellular matrix within amnio-chorionic membrane due to collagen degradation is one of the processes that predispose to PROM (4, 5). During pregnancy, the weakening of amniotic membrane is hypothesized to be caused by distention of the fetal membrane and also by certain biochemical processes that result in remodeling and apoptosis. Microscopic observation of fetal membrane in PROM cases shows decreased collagen tissue, disturbance of the collagen structure, and also increased collagenolytic activity (6-8). The degradation of collagen is caused by matrix metalloproteinase (MMP). The MMPs can be activated by the apoptosis sequences and apoptosis itself can also be induced by MMPs activation (9-11).

The signals that induce apoptosis may come from both intracellular and extracellular compartments: the extrinsic/extracellular pathway (initiated through the stimulation of death receptors) and the intrinsic pathway (initiated through the release of signals from intracellular mitochondria/mitochondrial pathway). Both the intrinsic and extrinsic pathways are caspase-dependent and end up at the same point: the execution phase, marked by the activation of caspase-3 as the initiator of apoptosis (12-14). Some types of cell death may occur without caspase activation, especially in conditions where the cell is affected by infection or stress. In this situation, apoptosis is likely to occur through caspase-independent pathways that involve B-cell lymphoma 2 (Bcl-2) pro-apoptosis family members known as Bax. This caspase-independent pathway is triggered by infection-associated deoxyribonucleic acid (DNA) fragmentation due to mitochondrial damage. When a cell is exposed to DNA-damaging agents, intracellular p53 protein is activated and its increase then triggers apoptosis through the release of Bcl-2 proteins. This condition further increases the permeability of the mitochondrial membrane, releasing other apoptosis proteins such as cytochrome C, Smac, DIABLO, Apoptosis Inducing Factor (AIF), and endonuclease G (15-17).

Although many studies on the proteins involved in apoptosis processes in PROM cases have been conducted, mostly focused only on the classic caspase-dependent pathways (18-20). As the result, the exact process by which apoptosis occurs in PROM remains unclear. To date, no study has investigated the role of caspase-independent pathways in the pathogenesis of PROM, especially the role of AIF as the main apoptosis protein involved in the pathway. Caspase-dependent activity can be measured by caspase-3 expression while the independent pathway by AIF expression. In this case-control study, we aim to evaluate the expression of caspase-3, AIF, and Bcl-2 as risk factors for PROM.

## 2. Materials and Methods

The case-control study was conducted at the Sanglah General hospital, Bali, Indonesia, from December 2016 until December 2017 in two groups. There was no difference in terms of age and body mass index (BMI) in participants. The case group (n = 21) were selected from women with singleton pregnancies at 20 to 42 wk gestation complicated by PROM, while the controls (n = 21) were women with uncomplicated deliveries. The exclusion criteria implemented in the study were: obesity (BMI at the first antenatal visit), maternal infection, other obstetrics complications (hypertension in pregnancy, gestational diabetes, etc.), repeat preterm premature rupture of the membrane, and maternal illness (maternal heart disease, autoimmune disease, etc). The diagnosis of PROM was made based on the clinical finding: spontaneous rupture of the membrane at least an hour before the onset of labor.

The participants had spontaneous vaginal delivery or through induced labor. After the delivery, tissue samples were taken from the edge of membrane rupture based on macroscopic examination. Immunohistochemistry examination was used to determine Caspase-3, AIF, and Bcl-2 expressions in the membrane. Cells that express the determined protein appear dark brown-stained with blue nucleus. In this study, the positive result was defined as being ≥ 10% of the cells that were stained. The examination was carried at the Integrated Biomedical Laboratory of the Medical Faculty, Udayana University, Bali, Indonesia.

### Ethical consideration

This study had been approved by the Ethics Committee of the Sanglah hospital/Udayana University (code: 187/UN.14.2/KEP/2016). Written informed consent was obtained from all participants prior to conducting the study.

### Statistical analysis

Normality testing using Shapiro-Wilk normality test was used to assess the data distribution (p > 0.05) between the case and control groups in terms of age, parity, and BMI. Homogeneity test was also used. Independent t test was used to analyze the differences between caspase-3, AIF, and Bcl-2 expressions between the two groups. The correlation between Caspase-3, AIF, and Bcl-2 expressions with PROM was assessed using the Chi-square test and are expressed in odds ratio (OR).SPSS software (Statistical Package for the Social Sciences, SPSS Inc, Chicago, Illinois, USA) version 21 for Windows was used for statistical analysis.

## 3. Results

Forty-two subjects (21 cases and 21 controls) were included in this study. The case group consisted of 9 (42%) preterm and 12 (58%) term pregnancies, whereas the control group consisted of 10 (47%) preterm and 11 (53%) term pregnancies. Independent *t* test showed no significant differences in parity, age, and BMI between the two groups (Table I). Normality testing using Shapiro-Wilk test showed a normal distribution between the case and control groups in terms of age (p = 0.525 vs. 0.293), parity (p = 0.102 vs. 0.087), and BMI (p = 0.109 vs. 0.041). Homogeneity test revealed homogenous characteristics between both groups (age, p = 0.984; parity, p = 0.137; and BMI, p = 0.432).

**Figure 1:**
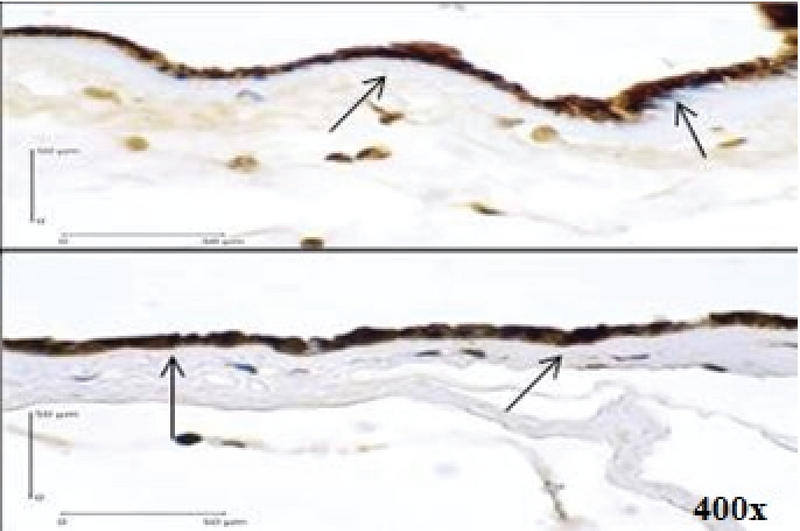
Positive Expression of Caspase-3 in Immunohistochemistry examination of the Amniotic Epithelial Cells (400x magnification) shown by dark brown cytoplasm staining with blue nucleus (arrows).

**Figure 2:**
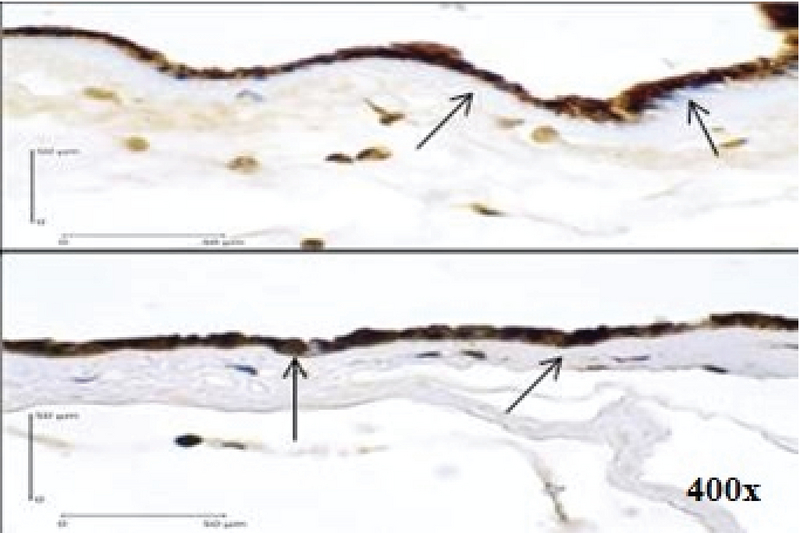
Positive Expression of AIF in Immunohistochemistry examination of the Amniotic Epithelial Cells (400x magnification) shown by dark brown cytoplasm staining with blue nucleus (arrows).

**Figure 3:**
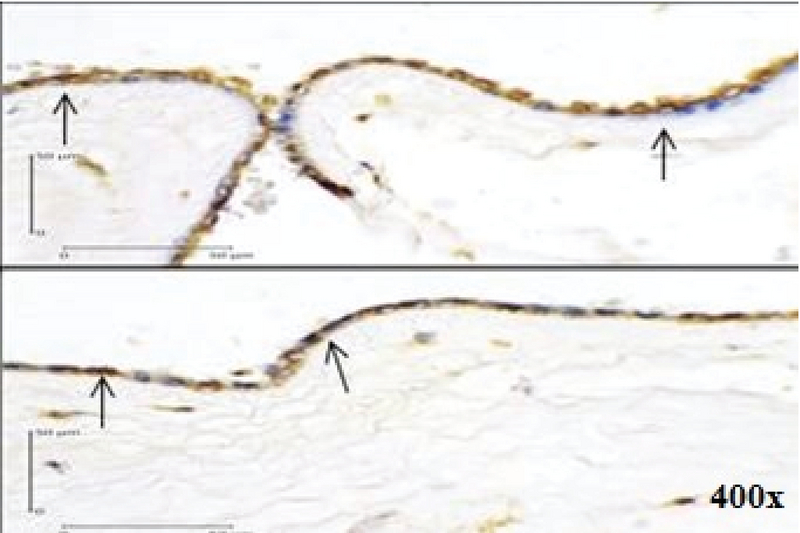
Positive Expression of Bcl-2 in Immunohistochemistry examination of the Amniotic Epithelial Cells (400x magnification) shown by dark brown cytoplasm staining with blue nucleus (arrows).

Using a Chi-square test to analyze the relationship between variables, we found a positive relationship between caspase-3 expression (Figure 1) and the increased risk of PROM by 9.75 times. Positive AIF expression (Figure 2) also significantly increased the risk of PROM by 6.60 times (Table II). The role of Bcl-2 anti-apoptosis protein expression (Figure 3) in PROM was also analyzed. We found that low expression of Bcl-2 was associated with an increased risk of PROM by 8.00 times (Table II).

**Table 1:** Distribution of demographic characteristics in two study groups


**Risk factor**	**Case group (n = 21)**	**Control group (n = 21)**	**p-value**
**Age (yr)**	27.67 ± 7.07	27.95 ± 7.29	0.89
**Parity**	0.52 ± 0.68	0.95 ± 1.28	0.18
**Body mass index (kg/m ${}^{2}$)**	24.63 ± 4.48	24.88 ± 3.87	0.85
Data presented as Mean ± Standard deviation. Chi square test			

**Table 2:** Risk of Preterm rupture of membrane based on caspase-3, Apoptosis Inducing Factor, and B-cell lymphoma 2 expressions in amniotic epithelial cells


**Expression**		**Groups**		**Odds ratio**	**95% CI**	**p-value**
	Case	**Control**		
**Caspase-3 **						
	**Positive**	18	8		
	**Negative**	3	13	9.75	2.16-43.95	0.001
**Apoptosis inducing factor**						
	**Positive**	18	10		
	**Negative**	3	11	6.60	1.48-29.36	0.009
**B-cell lymphoma 2 **						
	**Negative**	18	9		
	**Positive**	3	12	8.00	1.79-35.74	0.004
Data presented as number. Chi square test						

## 4. Discussion

The role of caspase-3 in the incidence of PROM was investigated by Xu and Wang (13). The study evaluated the role of caspase-3 expression and MMP activity at amniochorionic membrane in cases of PROM. Amniotic membranes were obtained from several groups of women: women with spontaneous PROM (n = 8), women with spontaneous vaginal delivery at term (n = 8), and women with uncomplicated repeated cesarean section (n = 8). The caspase-3 peptide was evaluated with immunohistochemistry. Positive caspase-3 expression was almost 2-3 times higher in the PROM group (mean expression 62.86 ± 3.83%), as compared with spontaneous vaginal delivery (42.33 ± 2.99%) and the cesarean section group(20.97 ± 2.94%) which indicated an increased level of apoptosis in the amniotic cells (p < 0.005) (13).

The mechanism of apoptosis through the caspase-independent pathway does not require caspase as a messenger with a specific role in the cell-death mechanism. This pathway is induced mainly by mitochondrial pro-apoptosis protein molecules, AIF and endonuclease G. Translocation of these two proteins from mitochondria to the nucleus will cause nuclear DNA fragmentation (15-17). AIF is regarded as one of the main protein that mediates caspase-independent cell death. Studies found that apoptosis cannot be activated by caspase alone. Cell death can even be induced without the involvement of caspase, through the expression of Bax (or Bak). This process involves mitochondrial factors, such as AIF, which induces mitochondrial swelling, chromatin condensation, and cytochrome C release without caspase activation (18, 19, 21).

The increased apoptosis at the trophoblast chorionic layer of term pregnancies was investigated by Harirah and colleagues. They found an increased apoptotic index on the chorionic trophoblast of the distal part of the ruptured amniotic membrane which was three times higher compared to the artificial rupture in cesarean section. The choriodecidual layer of spontaneous delivery showed higher pro-apoptotic activity (high caspase-3 and low Bcl-2), compared to cesarean delivery (22). The Bcl-2 protein family is a key regulator of apoptosis which regulates the permeability of the outer mitochondrial membrane and the release of pro-apoptotic factors. They are also a key regulator of apoptosis because they connect the extrinsic and intrinsic pathways (15, 23, 24).

Both the intrinsic and extrinsic pathways may induce the activation of caspase in PROM; however, the intrinsic pathway is more dominant in term pregnancies. Studies also reported significant differences between Bcl-2, caspase-3, and caspase-9 in the supracervical area, which represent the intrinsic pathway activity. Fas and its ligand, (Fas-L), were also found in all amniotic membrane samples but there was no significant increase at the supracervical and the distal membrane areas. Thus, it is hypothesized that the extrinsic pathway is not involved in the remodeling of the amniotic membrane. Even though pro-apoptotic protein Bax expression was not significantly different, the anti-apoptotic protein Bcl-2 expression was found to be significantly decreased at the paracervical area (25-27).

Caspase-3 is the main family member of caspase that is actively involved in apoptosis. The activation of caspase-3 is the biochemical reaction that occurs before the initiation of apoptosis, and almost all apoptosis proceeds via this pathway. Thus, investigating the expression of caspase-3 may indicate the activity of cellular apoptosis. Immunohistochemistry examination of the amniotic membrane in PROM cases shows expression of caspase-3 on the amniotic epithelial cells and chorionic cytotrophoblast cells, while its expression is limited in mesenchymal and the reticular cells of the matrix. This indicates that apoptosis occurs at the amniotic and chorionic layers, and it plays important role in the regulation of the fetal membrane (13, 25, 27).

There are two major apoptotic pathways in the PROMs that can be initiated by infection, genotoxic agents, or other unknown factors. The first pathway utilizes tumor necrosis factor receptor-1 and the Fas receptors. These receptor proteins bind to their specific ligands, tumor necrosis factor and Fas L, respectively, and initiate signal transduction through two adapter proteins: tumor necrosis factor receptor type 1-associated death domain protein and Fas-associated protein with death domain. These domain proteins activate a group of proteases known as caspases. They also independently activate procaspase-8 into caspase-8, subsequently enabling caspase-3 (20, 28).

The other pathway may be initiated by p53, by increasing transactivator p53 proteins, which causes mitochondrial membrane damage and release cytochrome C. This event will then activate apoptosis protease-activating factor -1, which converts procaspase-9 into its active form, which in turn initiates the activation of the effector caspase-3, -7, and -6. The result of this process is proteolysis of structural proteins, homeostasis proteins, and some other proteins, ultimately programming cell death (20). Pro-apoptotic proteins that are also released by mitochondria during apoptosis are AIF, the endonuclease G, and CAD. AIF and endonuclease G independently cause DNA fragmentation and chromatin condensation (29-31).

Our study found caspase-3 expression in amniotic epithelial cells, which indicates the apoptotic process in the amnion; its expression was higher in PROM cases by 9.75 times. This suggests that caspase-3 expression is a risk factor for the PROM. Caspase-3 is an executor caspase that acts in the caspase-dependent, extrinsic and intrinsic pathways of apoptosis, which explains its dominant expression in apoptosis. Negara found that positive caspase-3 expression increased the risk of PROM by 7.3 times (OR 7.31; 95% CI 2.64-20.22; p = 0.001) (32). These findings indicate that caspase-dependent pathway of apoptosis plays a central role in the mechanism of PROM.

In the present study, low Bcl-2 expression was associated with an increased risk of PROM by eight times. This suggests that Bcl-2 is an anti-apoptotic protein that acts as a regulator of apoptosis, and its low level is a risk factor for PROM. Furthermore, the current study also found that high AIF expression was associated with an increased risk of PROM by 6.60 times. The previous study by Negara found that positive AIF expression is a risk factor of PROM by 5.10 times (OR5.10;95% CI 1.86-13.96; p = 0.001). AIF expression was more profound in the PROM group. It is a pro-apoptotic protein released by mitochondria in the caspase-independent pathway. Thus, high AIF expression indicates the involvement of caspase-independent pathway in PROM (33).

Our findings suggest that apoptosis via the caspase-dependent and caspase-independent pathways may be involved in PROM. It is evident that the high caspase-3 and AIF expressions and low Bcl-2 expression are significant risk factors that play important roles in the mechanism of PROM. This would imply that apoptosis mechanism in PROM may not be so simple. It may be comprised of various initiation processes and does not depend on only a specific molecular mechanism. This may explain the difficulty of predicting which pregnancy is more vulnerable to PROM. A complete understanding on the underlying process in PROM may assist clinician to apply preventive measures on high-risk pregnancies. Further studies are needed in the future to better define the complete process.

## 5. Conclusion

We found significant correlations between caspase-3, AIF, and Bcl-2 expressions with PROM. High expressions of caspase-3 and AIF were significant risk factors for the occurrence of PROM in term and preterm pregnancy. Low expression of Bcl-2 was also associated with the increased risk of PROM. Among the three variables, caspase-3 expression was the most dominant risk factor of PROM. We can conclude that apoptosis was a key component in the mechanism of PROM and that both caspase-dependent and caspase-independent apoptotic pathways were involved in PROM.

##  Clinical Significance

Apoptosis process in PROM was thought to be mediated by a caspase-dependent pathway. This study shows that the caspase-independent pathway is also involved in PROM.

##  Conflict of Interest

The authors declare no conflict of interest regarding the publication of this paper.
